# Indeterminate and discrepant rapid HIV test results in couples' HIV testing and counselling centres in Africa

**DOI:** 10.1186/1758-2652-14-18

**Published:** 2011-04-08

**Authors:** Debrah I Boeras, Nicole Luisi, Etienne Karita, Shila McKinney, Tyronza Sharkey, Michelle Keeling, Elwyn Chomba, Colleen Kraft, Kristin Wall, Jean Bizimana, William Kilembe, Amanda Tichacek, Angela M Caliendo, Eric Hunter, Susan Allen

**Affiliations:** 1 Emory Vaccine Center at Yerkes National Primate Research Center, Emory University, Atlanta, Georgia, USA; 2 Department of Pathology and Laboratory Medicine, Emory University School of Medicine, Atlanta, Georgia, USA; 3 Rollins School of Public Health, Department of Global Health, Emory University, Atlanta, Georgia, USA; 4 Emory Center for AIDS Research, Emory University, Atlanta, Georgia, USA; 5 Projet San Francisco, Kigali, Rwanda; 6 Zambia Emory HIV Research Project, Lusaka, Zambia

## Abstract

**Background:**

Many HIV voluntary testing and counselling centres in Africa use rapid antibody tests, in parallel or in sequence, to establish same-day HIV status. The interpretation of indeterminate or discrepant results between different rapid tests on one sample poses a challenge. We investigated the use of an algorithm using three serial rapid HIV tests in cohabiting couples to resolve unclear serostatuses.

**Methods:**

Heterosexual couples visited the Rwanda Zambia HIV Research Group testing centres in Kigali, Rwanda, and Lusaka, Zambia, to assess HIV infection status. Individuals with unclear HIV rapid antibody test results (indeterminate) or discrepant results were asked to return for repeat testing to resolve HIV status. If either partner of a couple tested positive or indeterminate with the screening test, both partners were tested with a confirmatory test. Individuals with indeterminate or discrepant results were further tested with a tie-breaker and monthly retesting. HIV-RNA viral load was determined when HIV status was not resolved by follow-up rapid testing. Individuals were classified based on two of three initial tests as "Positive", "Negative" or "Other". Follow-up testing and/or HIV-RNA viral load testing determined them as "Infected", "Uninfected" or "Unresolved".

**Results:**

Of 45,820 individuals tested as couples, 2.3% (4.1% of couples) had at least one discrepant or indeterminate rapid result. A total of 65% of those individuals had follow-up testing and of those individuals initially classified as "Negative" by three initial rapid tests, less than 1% were resolved as "Infected". In contrast, of those individuals with at least one discrepant or indeterminate result who were initially classified as "Positive", only 46% were resolved as "Infected", while the remainder was resolved as "Uninfected" (46%) or "Unresolved" (8%). A positive HIV serostatus of one of the partners was a strong predictor of infection in the other partner as 48% of individuals who resolved as "Infected" had an HIV-infected spouse.

**Conclusions:**

In more than 45,000 individuals counselled and tested as couples, only 5% of individuals with indeterminate or discrepant rapid HIV test results were HIV infected. This represented only 0.1% of all individuals tested. Thus, algorithms using screening, confirmatory and tie-breaker rapid tests are reliable with two of three tests negative, but not when two of three tests are positive. False positive antibody tests may persist. HIV-positive partner serostatus should prompt repeat testing.

## Background

Sub-Saharan Africa remains the focal point of the HIV pandemic, with the largest percentage of HIV-positive individuals and the greatest number of new infections per year [1]. Most new infections in this region occur through heterosexual transmission in cohabiting discordant couples where one partner is HIV positive and the other is uninfected [2-5]. It is striking that 40% to 50% of cohabitating HIV-infected individuals in east Africa have an HIV-uninfected partner [6], and yet most do not know their own or their partner's status, resulting in an estimated transmission rate among uncounselled discordant couples of 12% to 20% per year [3,7-9].

Couples' voluntary counselling and testing (CVCT) is a proven HIV prevention strategy for cohabiting couples [7,10,11]. Studies have shown that counselled couples are more likely to use condoms and less likely to acquire HIV or sexually transmitted infections (STIs) [5,12,13]. CVCT centres offering same-day rapid antibody testing are of particular value in resource-limited settings where distance and costly transportation limits access to services [4,14-16].

The HIV testing strategies and relevant national HIV testing algorithms of the Centers for Disease Control and Prevention (CDC), the Joint United Nations Programme on HIV/AIDS (UNAIDS) and World Health Organization (WHO) recommend the sequential or parallel use of two to three different HIV antibody assays [17]. Rapid HIV tests come in ready-to-use kits, which require no additional reagents or special equipment, and are reported to detect all subtypes in Africa with similar sensitivity and specificity. Most assays can be completed in a few easy steps, giving visual results in less than 20 minutes. High sensitivity tests are preferred for screening, while confirmatory tests ideally have high specificity.

When the results of the screening and confirmatory tests are not the same (discrepant), or any given test yields unclear results (indeterminate), the HIV infection status of the individual may be determined through use of additional tests. These may include a third rapid test as a tie-breaker, an enzyme-linked immunosorbent assay (ELISA) test for detection of antibodies and/or antigen, and HIV-RNA viral load testing [18-20]. Reported causes of indeterminate or discrepant rapid test results include early HIV infection [19,21-24] and false positive reactions due to malaria, pregnancy, syphilis, hepatitis B or endemic infections [25-29].

As the likelihood of early infection is highest in HIV-discordant couples [3,10,15,30], we present the results of an algorithm using three serial rapid HIV tests in cohabiting couples and describe performance of the algorithm in two cities, with two primary circulating subtypes, in central (Kigali, Rwanda, subtype A) and southern (Lusaka, Zambia, subtype C) Africa.

## Methods

### Study participants

Testing and counselling occurred at the Rwanda-Zambia HIV Research Group (RZHRG) couples' voluntary counselling and testing (CVCT) centres in Kigali, Rwanda, and Lusaka, Zambia. Promotion and counselling procedures have been detailed elsewhere [7,10,15].

### HIV rapid antibody assays

Venipuncture blood samples obtained from CVCT study participants were sequentially tested with rapid HIV antibody qualitative assays (rapid tests). The four assays used included: Determine HIV-1/HIV-2™ (sensitivity 100%, specificity 99.7%) (Abbott Laboratories, Abbott Park, IL) or First Response^® ^HIV Card Test 1-2.0 (sensitivity and specificity, 100%) (Premier Medical Corporation Ltd., Colonia, NJ) for screening, and Capillus HIV-1/HIV-2 (sensitivity 100%, specificity 99.7%) (Trinity Biotech, Ireland) and Uni-Gold™ HIV (sensitivity and specificity, 100%) (Trinity Biotech, Ireland) for confirmatory and/or tie-breaker testing.

All assays detect antibodies to HIV-1 and HIV-2, and were performed according to the manufacturers' protocols and the RZHRG standardized operating procedure. In general, 10-60 μl of plasma was applied to the sample pad and visually read as per manufacturer's instructions at the required time, three to 15 minutes later. Routine standard operating procedure (SOP) trainings and quality assurance programmes are provided to technicians.

An unambiguous band in the sample window was indicative of a positive result with First Response, Determine and Unigold. No band in the sample window was scored as negative. With the Capillus agglutination test, the presence of a white aggregate with a clear background in the viewing window was scored as positive and lack of any agglutination was scored as negative. If a result could not be clearly determined by the trained technician, such as a faint band or small milky white agglutinated particles, the same test was rerun and two technicians read both tests. The laboratory manager performed the final quality control on all final results.

These results were read only in the presence of a proper positive control as per manufacturers' protocol. As an additional step, quality control was performed at the beginning of each work day and with each newly opened kit.

### HIV testing algorithm for couples

The HIV testing algorithm used was adapted from WHO [17], and influenced by guidelines in Rwanda and Zambia over time and by availability of test kits provided by the national HIV testing programmes [31].

**Figure 1:**
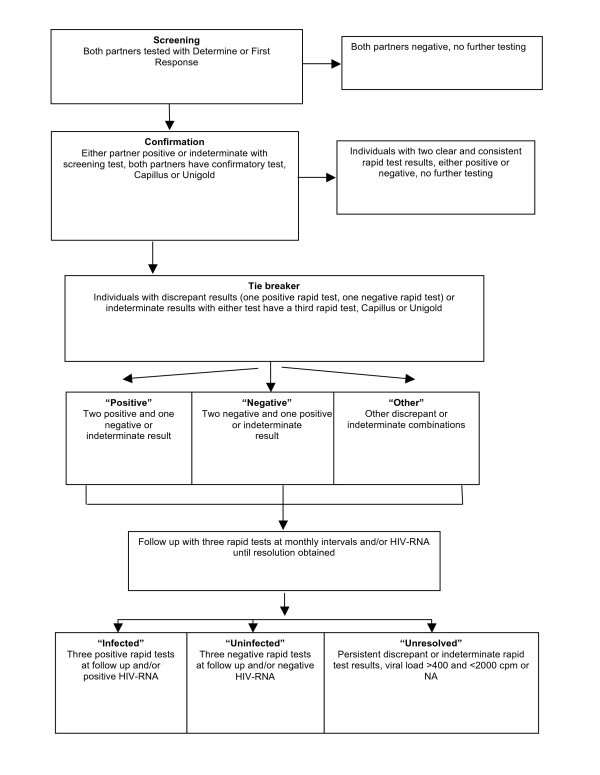
**Rwanda Zambia HIV Research Group HIV Testing Algorithm**. Couples visiting RZHRG voluntary counselling and testing centres were serially tested with four possible HIV rapid tests. Discrepant results were identified as one positive and one negative result. Results not clearly positive (weak band or poor agglutination) were classified as indeterminate. Discrepant and/or indeterminate individuals were asked to return for repeat monthly testing. HIV-1 RNA RT-PCR was performed on more challenging cases.

Figure 1 describes the use of four possible rapid tests for screening, a confirmatory test and a tie-breaker where necessary. All samples were initially tested, only once, with one of two possible screening tests (Determine or First Response) depending on availability of kits in country. Couples where both partners had a negative screening test were counselled as HIV negative and the couple was not followed further.

In couples where either partner had either a positive or indeterminate screening result, both partners were given a confirmatory test. If either partner now had two clearly positive tests, the individual concerned would be counselled as positive; if either partner had two clearly negative tests, he or she would be counselled as negative. In the event that the screening or confirmatory test result was indeterminate or one of the tests had different results (discrepant), a third test was used as a tie-breaker for the individual concerned. The individual was counselled as positive, negative or indeterminate/discrepant based on the results from two of the three possible tests (Two of Three rule), and also asked to return in one month for repeat testing with all three rapid tests on each return visit in order to resolve his or her serostatus. Monthly follow up continued until the infection status was clear.

### RZHRG HIV testing classifications for initial and follow-up testing

Individuals with indeterminate and/or discrepant HIV test results requiring monthly follow up were initially classified at their first visits using the Two of Three rule based on the three rapid test results. At the first visit, individuals where two of three rapid tests were clearly positive and the third either indeterminate or negative were initially classified as "Positive"; those with two of three tests clearly negative and the third either positive or indeterminate were classified "Negative"; and those with any other combination, including two discrepant and/or indeterminate results, were classified as "Other".

"Positive", "Negative" and "Other" individuals were given an indeterminate/discrepant counselling message based on their initial classification and asked to return. At the follow-up visit, all three rapid tests were again performed (screening, confirmatory and tie-breaker tests). If repeat testing showed clear and consistent results with all three rapid tests, the case was resolved as either HIV infected (three positive tests) or HIV uninfected (three negative tests). If repeat testing did not show clear consistent results with all three rapid tests, the individual was counselled based on the Two of Three rule and asked to return for follow up. Whereas the initial classification and possible follow-up visits were based on two of three tests, all three tests had to be consistent for a "Final Resolution" to be determined.

If indeterminate/discrepant ("D") results persisted for longer than two months or if no follow-up samples were available, quantitative, non-diagnostic, HIV-1 RNA RT-PCR (Amplicor HIV-1 Monitor Test, v1.5, standard version, Roche Diagnostics, Indianapolis, IN) was performed. HIV-RNA viral loads of less than 400 copies/mL (the lower limit of detection of the test) were considered "Uninfected" and a HIV-RNA viral load of >2000 copies/mL was considered "Infected". Because the Roche HIV-1 RNA RT-PCR assay is deemed non-diagnostic, in a conservative effort, the cut-off for resolving "Infected" cases was >2000 copies/mL. The few cases where the HIV-RNA viral loads were between 400 and 2000 copies/mL were not used to resolve final infection status. Patient follow up was only up to three months and if HIV-RNA viral load could not be used for final resolution, the infection status remained unknown in this study.

### Data analysis

Data were analyzed using the SAS software package (version 9.2; SAS Institute, North Carolina, USA). Frequency distributions and cross-tabulations were generated comparing the Two of Three and Final Resolution results, stratified by city and partner HIV status. Proportions were compared using Chi-square tests, with Fisher's exact test used when any value was less than five.

## Results

### Initial HIV classifications

From 1 August 2005 to 30 March 2007, 12,952 couples were tested at the Projet San Francisco CVCT centres in Kigali, Rwanda. From 1 January 2002 to 30 March 2008, 9958 couples were tested at the Zambia-Emory HIV Research Project in Lusaka, Zambia.

Of the total of 22,910 couples (45,820 individuals) tested at the two sites: 14,689 (64%) couples were concordant negative (male, M-:female, F-), 4250 (19%) couples were concordant positive (M+:F+) and 3034 (13%) couples were discordant (M+:F- and M-:F+). In addition, 937 (4%) couples had indeterminate and/or discrepant or incomplete test results (Table 1). Of the 937 couples involved, overall, 1045 individuals had indeterminate, discrepant or incomplete test results after the three rapid tests during the first testing opportunity. In some couples both partners were affected, therefore 1045 individuals were concerned, but in total only 937 couples. These 1045 individuals were asked to return for repeat testing to resolve their HIV status.

**Table 1:** Initial HIV classifications based on rapid antibody test results from two CVCT centres

	Kigali, Rwanda		Lusaka, Zambia		Total	
	**August 1, 2005 - March 30, 2007**		**January 1, 2002 - March 30, 2008**			
	**N**	**%**	**N**	**%**	**N**	**%**
Total couples tested	12,952		9,958		22,910	
Clear concordant						
M- : F-	10,122	78.15%	4,567	45.86%	14,689	64.12%
M+ : F+	712	5.50%	3,538	35.53%	4,250	18.55%
Clear discordant						
M+ : F-	584	4.51%	811	8.14%	1,395	6.09%
M- : F+	733	5.66%	906	9.10%	1,639	7.15%
Subtotal couples with clear results	12,151	93.82%	9,822	98.63%	21,973	95.91%
Indeterminate/Discrepant (D) rapid test results						
M- : FD	242	1.87%	25	0.25%	267	1.17%
MD : F-	387	2.99%	50	0.50%	437	1.91%
MD : FD	102	0.79%	6	0.06%	108	0.47%
MD : F+	44	0.34%	42	0.42%	86	0.38%
M+ : FD	26	0.20%	13	0.13%	39	0.17%
Subtotal with at least one partner having unclear results	801	6.18%	136	1.37%	937	4.09%

Initial classifications for couples tested between Kigali, Rwanda, and Lusaka, Zambia, were clearly concordant negative (both partners HIV negative, M-:F-), clearly concordant positive (both partners HIV positive, M+:F+), clearly discordant (one partner negative, one partner positive; M+:F-, M-:F+). Couples classified as indeterminate and/or discrepant (D) either had one partner clearly negative (M-:FD, MD;F-), one partner clearly positive (MD:F+, M+:FD), or both partners indeterminate and/or discrepant (MD:FD).

Twenty-three individuals of these 1045 lacked three rapid test results at initial testing and were not included in further analysis. Of the remaining 1022 individuals, 361 (35%) did not return for follow up and their remaining samples could not be assessed with HIV-RNA testing. The proportion of individuals who did not return for follow up was higher for those who were initially classified as "Negative" (39%) than for those who were classified as "Positive" or "Other" (28% and 27%, respectively) (data not shown). These individuals were not included in further analysis. This resulted in 661 individuals in this study who were followed with repeat testing to resolve their HIV status.

In this study, the overall prevalence of HIV was lower in the Kigali cohort compared with the Lusaka cohort, with similar prevalence in men and women. In Kigali, 6% of couples were concordant positive, 10% were discordant, and at least 6.2% had one partner with unclear results. At the individual level, this resulted in 5% of males and 6% of females being HIV positive. In Lusaka, 36% of couples were concordant positive, 17% discordant, and at least 1.4% had with unclear results. At the individual level, this resulted in 22% of males and 23% of females being HIV positive.

Interestingly, the prevalence of individuals with indeterminate and/or discrepant results was comparatively higher in Kigali (3.5% of individuals vs. 0.7% in Lusaka). In both cities, men were more likely to have indeterminate/discrepant results than women: 59% (533 of 903 individuals with such results) in Kigali and 69% (98 of 142 individuals) in Lusaka were men.

### Two of Three rule

Table 2 shows initial classifications for individuals with indeterminate/discrepant profiles based on the Two of Three rule. Individuals who initially had two of three negative test results were initially classified as "Negative" [62% (410/661)]; those with two of three positive tests were initially classified as "Positive" [6% (37/661)]; and those with two of three indeterminate or discrepant tests were initially classified as "Other" [32% (214/661)]. These initial classifications are further divided into their final resolution after repeat rapid testing and/or HIV-RNA testing.

**Table 2:** Individual initial HIV classifications by Two of Three rule and final resolutions

	Kigali, Rwanda				Lusaka, Zambia				Total			
	**Final Resolution**				**Final Resolution**				**Final Resolution**			
	**Total**	**Uninfect**	**Infect**	**Unresolv**	**Total**	**Uninfect**	**Infect**	**Unresolv**	**Total**	**Uninfect**	**Infect**	**Unresolv**
Two of Three Negative	354	219	4	131	56	46	0	10	410	265	4	141
		62%	1%	37%		82%	0%	18%		65%	1%	34%
Two of Three Positive	23	14	8	1	14	3	9	2	37	17	17	3
		61%	35%	4%		21%	64%	14%		46%	46%	8%
Two of Three Other	201	128	5	68	13	8	5	0	214	136	10	68
		64%	2%	34%		62%	38%	0%		64%	5%	32%

Individuals with initial two of three rapid tests negative ("Two of Three Negative") were classified as "Negative"; those with initial two of three rapid tests positive ("Two of Three Positive") were classified as "Positive". Individuals with two of three rapid test results indeterminate/discrepant ("Two of Three Other") were classified as "Other". All were asked to return for repeat testing and final resolution. Individuals with final three of three rapid test results negative were resolved as HIV uninfected ("Uninfected"); those with three of three rapid test results positive were resolved as HIV infected ("Infected"). Individuals with persistent indeterminate/discrepant rapid test results were finally resolved as "Unresolved".

The table in Additional file 1 shows the frequency distribution of combinations of individuals' rapid test results by the initial Two of Three classification. Among 410 samples initially classified as "Negative" based on the Two of Three rule, 383 (93.4%) had positive or indeterminate screening test results (Determine or First Response) and both negative confirmatory and tie-breaker tests (Unigold and/or Capillus). In 18 (4.4%) cases, the Unigold was positive or indeterminate, and in nine (2.2%), the Capillus was positive or indeterminate.

In the 214 samples in the "Other" group, the First Response was positive or indeterminate in 28 (13%) and 79 (37%) cases, respectively. Corresponding numbers for Determine were 24 (11%) positive and 78 (36%) indeterminate; for Unigold, 18 (8%) positive and 120 (56%) indeterminate; and for Capillus, 24 (11%) positive and 113 (53%) indeterminate.

Of the 37 samples in the "Positive" group, 26 (70%) were positive with a screening test (nine First Response, 17 Determine), 28 (76%) with Capillus, and 21 (57%) with Unigold. Overall, screening tests were more likely to be positive than confirmatory tests, and with all tests, indeterminate results were more common than discrepant results.

### Final resolutions

Overall, 63% (418/661) of individuals who had indeterminate or discrepant results at the first testing opportunity were subsequently resolved as "Uninfected" and 32% (212/661) as "Unresolved" (maintained indeterminate or discrepant HIV test results on re-testing at intervals of a month or more, up to three months). Only 5% of individuals (31/661) were resolved as "Infected".

#### Initial classification "Negative"

The majority (65%) of those initially classified as "Negative" (n = 410) based on the Two of Three rule were resolved as "Uninfected", and 34% remained "Unresolved" but did not seroconvert during the three months of follow up, although the indeterminate/discrepant serologic pattern persisted. Only 1% (4/410) of individuals seroconverted two, five, six and 14 months after the first test (Table 2, Table 3). All four cases were in Kigali. In two individuals, the HIV-RNA viral load was undetectable at the time of the initial test and later became positive, suggesting that the infection was likely unrelated to the initially discrepant/indeterminate rapid test result. The proportion of individuals initially classified as "Negative" resolving as "Uninfected" was lower in Kigali (62%) than in Lusaka (82%). More Kigali individuals maintained their indeterminate/discrepant serologic patterns without seroconverting (37% in Kigali vs. 18% in Lusaka, p = 0.01).

**Table 3:** Complex cases

Case Study	Country	Sex	Visit Date	Partner Status	Rapid Test Results			Viral Load
**Two of three Negative, resolved as HIV Infected**					**Determine/** **First Response**	**Capillus**	**Unigold**	
Case A	R	M	28-Sep-2005	POS	D	-	-	
			12-Oct-2005		+	-	-	
			23-Jan-2006		+	-	-	0
			22-Feb-2006		+	-	-	
			22-Mar-2006		+	-	-	
			11-Apr-2006		+	-	-	
			11-Jul-2006		+	-	-	
			16-Oct-2006		+	D	D	
			16-Nov-2006		+	D	+	
			24-Nov-2006		+	+	+	
			27-Nov-2006		+	+	+	636
			14-Feb-2007		+	A	+	28100
Case B	R	M	9-Apr-2006	POS	-	-	D	
			19-Apr-2006		-	-	-	
			18-Jul-2006		-	A	A	0
			17-Oct-2006		-	-	D	
			25-Oct-2006		+	+	+	63100
Two of three Positive, resolved as HIV Uninfected								
Case C	R	M	16-Oct-2005	NEG	+	+	-	
			20-Nov-2005		+	+	-	
			4-Jan-2006		+	D	-	0
Case D	R	M	18-Nov-2005	NEG	+	+	D	0
			7-Dec-2005		-	+	D	
			9-Jan-2006		-	+	+	
			9-Feb-2006		-	+	D	
			13-Mar-2006		-	+	D	427
			5-Dec-2006		-	+	+	930
			12-Sep-2007		-	-	D	
			15-Oct-2007		-	+	D	
			7-Dec-2007		-	-	-	0
			7-Mar-2008		-	D	D	
Two of three Negative, Unresolved								
Case E	Z	M	22-Sep-2005	POS	+	-	-	
			26-Oct-2005		D	-	-	
			30-Nov-2005		+	-	-	
			4-Jan-2006		D	-	-	1880
Case F	R	M	8-Feb-2006	POS	-	-	D	
			3-Mar-2006		-	-	+	
			3-Apr-2006		-	-	D	
			12-Jun-2006		-	-	+	1525
			31-Aug-2006		-	A	A	
			30-Nov-2006		-	-	+	
			6-Mar-2007		-	-	+	
			8-Jun-2007		-	-	+	
			3-Sep-2007		-	-	+	
			29-Nov-2007		-	-	+	
			3-Mar-2008		-	-	+	
			2-Jun-2008		-	-	+	
			1-Sep-2008		-	-	+	
Case G	R	F	26-Feb-2006	NEG	D	-	-	
			30-Mar-2006		D	-	-	
			5-May-2006		-	D	-	631
			23-May-2008		D	-	-	
Two of three Other, resolved as HIV Uninfected								
Case H	R	F	28-Apr-2006	DOUBTFUL	D	D	D	0
			2-Jun-2006		D	-	D	976
			23-May-2008		D	-	+	
			20-Jun-2008		-	D	-	
			24-Jul-2008		-	-	D	
Case I	R	M	2-Feb-2006	DOUBTFUL	D	D	D	
			2-Mar-2006		D	D	+	
			4-Apr-2006		D	D	D	
			12-May-2006		D	D	-	1129
			15-May-2008		-	-	D	
			20-Jun-2008		-	D	D	
			24-Jul-2008		-	-	-	

Eight cases (A-I) represent a subset of complex case studies for which the initial classifications, based on the Two of Three rule, and final resolutions differed. Final

resolutions were based on repeat rapid test results and/or HIV-RNA detection (HIV-RNA viral load). Clearly positive result (+); clearly negative result (-); indeterminate result (D); absent result (A).

#### Initial classification "Other"

Those initially classified as "Other" proved for the most part to be "Uninfected" (136/214) in both Kigali (64%) and Lusaka (62%). In Kigali, only 2% (5/201) resolved as "Infected" compared with 38% (5/13) in Lusaka. Individuals were more likely to be "Unresolved" in Kigali (persistent indeterminate or discrepant rapid test results) than in Lusaka (34% vs. 0%, p <0.001).

#### Initial classification "Positive"

Unexpected results were found in those who were initially classified as "Positive". Overall, 17 of 37 individuals resolved as "Infected", but 17 resolved as "Uninfected" and three remained "Unresolved". Again, the resolution of this group differed between Kigali and Lusaka, with only 35% (8/23) of individuals resolving as "Infected" in Kigali compared with 64% (9/14) in Lusaka (p <0.0001).

### Partner HIV status

Table 4 describes the correlation between an individual's final status resolution and partner HIV status. As expected, the partner's HIV status played a strong predictive role, with 48% (15/31) of indeterminate/discrepant cases who resolved as "Infected" having HIV-infected partners compared with 11% (44/418) of those who resolved as "Uninfected" and 5% (10/212) of those who remained "Unresolved" (p <0.0001). In Kigali, six of 17 individuals with indeterminate/discrepant results who eventually resolved as "Infected" had HIV-infected partners, compared with nine of 14 in Lusaka (p = NS).

**Table 4:** Final HIV resolutions classified by partner HIV status

	Final Resolution				Final Resolution				Final Resolution			
	**Kigali**				**Lusaka**				**Total**			
	**Uninfect**	**Infect**	**Unresolv**	**Total**	**Uninfect**	**Infect**	**Unresolv**	**Total**	**Uninfect**	**Infect**	**Unresolv**	**Total**
Partner HIV status												
Negative	244	9	146	399	44	3	7	54	288	12	153	453
%	61%	2%	37%		81%	6%	13%		64%	3%	34%	
Positive	33	6	7	46	11	9	3	23	44	15	10	69
%	72%	13%	15%		48%	39%	13%		64%	22%	14%	
Other/Unresolv	84	2	47	133	2	2	2	6	86	4	49	139
%	63%	2%	35%		33%	33%	33%		62%	3%	35%	
Total	361	17	200	578	57	14	12	83	418	31	212	661
%	62%	3%	35%		69%	17%	14%		63%	5%	32%	
% with HIV+ partners	9%	35%	4%		19%	64%	25%		11%	48%	5%	

Initially classified indeterminate/discrepant individuals were resolved as "Uninfected" or "Infected". "Unresolved" are further classified by partner's HIV status ("Negative", "Positive", "Other/Unresolved").

Of cases with a final resolution of "Infected" or "Uninfected", most (267, 59%) were resolved by repeat rapid testing at follow-up visits, with the remainder resolved by HIV-RNA testing (171, 38%) or both repeat antibody testing and HIV-RNA testing (11, 3%).

### Complex cases

Table 3 illustrates the complexities of the cases for which the initial classification and final resolutions differed. These include two of the four individuals initially classified as "Negative" and who eventually resolved as "Infected"; three "Negative" individuals by initial classification who remained "Unresolved"; and two "Positive" initial classifications who resolved as "Uninfected". A selection of two "Other" individuals who were resolved as "Uninfected" and had HIV-RNA viral loads between 400 and 2000 copies/mL are also shown.

In the two cases initially classified as "Negative" and resolved as "Infected", the long delay between the initial indeterminate/discrepant results, combined with undetectable HIV-RNA viral loads at those time points, suggests that the initial rapid test results may have been unrelated to the subsequent infections. Both of those individuals had HIV-infected partners and were likely to have had regular exposure and opportunity for transmission.

Of the 17 who were initially "Positive" and resolved as "Uninfected" (Table 2), 16 had undetectable HIV-RNA viral loads (Additional file 1) and one had low positive HIV-RNA viral loads on two occasions bracketed by undetectable HIV-RNA viral loads. They also eventually had three negative rapid tests (Table 3). Low positive HIV-RNA viral loads were found in four cases classified initially as "Negative". These were not interpreted as indicative of HIV infection given the lack of seroconversion in the months after follow up, and were classified as "Unresolved". Of the two examples of "Others" that resolved as "Uninfected", both had low HIV-RNA viral loads but did not seroconvert, and both their partners had indeterminate and/or discrepant test results (Table 3).

## Discussion

Rapid HIV testing algorithms using sequential or parallel testing are widely used in Africa [20,31]. In this study, a sequential testing algorithm was adapted for use in couples by adding a confirmatory test for both partners if either partner had a positive or indeterminate screening test. Of the 22,910 couples tested at two large CVCT sites in Kigali, Rwanda, and Lusaka, Zambia, 96% were provided clear results at their initial visits in which each partner had a final diagnosis resolved. The remaining 4% of couples included at least one partner with an indeterminate and/or discrepant HIV rapid test result (in total, 1045 individuals were concerned).

Thirty-four percent of individuals with an initial indeterminate or discrepant result did not return for follow up. The majority who did not return were initially classified as "Negative", perhaps suggesting that these individuals were complacent with their initial message. While data suggest that the majority of these individuals would have likely resolved as uninfected, one suggestion to increase follow up is a better designed counselling message that includes information pertaining to partner risk status. In addition to the 361 (34%) individuals who did not return for follow up, 212 of the 661 individuals who had repeat testing remained unresolved within the three months of follow up in this study. This resulted in a total of 573 individuals out of 45,820 (1.3%) not having access to a final diagnosis.

Of those 661 individuals with indeterminate/discrepant HIV rapid test results who returned for follow up, only 5% proved to be HIV infected, and half of these had HIV-infected partners. The Two of Three rule had good predictive value when two of three initial tests were negative (more than 99% uninfected), but not when two of three initial tests were positive (only 46% infected). Most individuals (64%) who could not be initially classified using two of three test results also proved to be HIV uninfected.

The frequency, distribution and resolution of indeterminate or discrepant rapid test results differed substantially in Kigali and Lusaka. The results presented here indicate that follow-up testing is generally not necessary for individuals with two negative tests and a negative partner. Individuals with other combinations of three rapid test results, including those with two positive results, should return in one month for follow-up testing and should not be assumed to be seroconverting.

### "Negative"

The most common indeterminate/discrepant profile was two negative and one positive or indeterminate rapid test result, noted in 62% of individuals. The majority (65%) of these individuals were resolved as HIV uninfected. Of the four individuals who did prove to be infected, two did not develop antibodies until six and 12 months after their first tests, and both had undetectable HIV-RNA viral loads prior to development of antibodies. If the indeterminate/discrepant results are considered unrelated to the subsequent infection, then only 0.5% of people with this profile were in the early infection period.

### "Other"

In 32% of individuals two out of three initial rapid tests were indeterminate and/or discrepant. Therefore these individuals could not be classified at their first visit as either "negative" (two out of three results negative) or "positive" (two out of three results positive). The majority of these individuals (64%) resolved as uninfected during follow up. Of the 10 (5%) who did prove to be infected, seven of eight who had follow up had seroconverted at their first follow-up visit, confirming that most individuals do not require prolonged follow up.

### "Positive"

Thirty-seven out of 661 (5.6%) individuals with indeterminate/discrepant rapid test results had two positive results and one negative or indeterminate result at their initial visit. Surprisingly, after follow-up testing, only 17 proved to be infected. This confirms that use of the Two of Three rule is not reliable when two of three results are positive and may be considered detrimental to individuals who have been falsely counselled as HIV positive. Programmes that use tie-breaker tests, as recommended by the Centers for Disease Control and Prevention in their HIV Rapid Test Training http://wwwn.cdc.gov/dls/ila/hivtraining/, must request follow-up testing to confirm infection.

### "Partner results"

Only 31 of 661 (5%) individuals with indeterminate/discrepant rapid test results at the first testing opportunity were later confirmed as HIV infected, and 15 (48%) of these had HIV-positive partners. In contrast, only 11% of individuals who were confirmed as HIV uninfected and 5% of individuals whose infection status was unresolved had HIV-positive partners. Partner testing should be encouraged whenever possible to maximize risk reduction and prevention impact [5,13,32-34]. Positive partner's serostatus is a useful indicator of HIV infection risk [3,35], and can facilitate the management and interpretation of indeterminate/discrepant rapid test results.

### "Persistent profiles"

One-third of indeterminate/discrepant individuals followed in this study maintained indeterminate/discrepant serologies at follow-up testing; in some cases, these results persisted for a year or more without seroconversion. These cases are a challenge to manage in a voluntary counselling and testing (VCT) setting. Some cases may have been due to delayed development of antibodies to HIV [21,22,36,37] or transient infection, which has been reported in infants [38-40]. Early or transient HIV infection is unlikely to be the explanation for the 95% (201) of individuals with this profile who had HIV-uninfected partners. Most of these responses were likely due to persistent false positive serologies from cross-reacting antibodies from intercurrent infection with other pathogens [26-29] or environmental exposure to test kit components, such as bovine products [41]. Where true infection is suspected, confirmatory testing for HIV-RNA should be considered when clear seroconversion does not occur after three to six months of follow-up testing [42-44]. Our study shows that in most cases a prolonged follow up is not needed.

### "Kigali vs. Lusaka"

Differences between Lusaka and Kigali emerged in prevalence of HIV, persistence of an indeterminate or discrepant test result, and how predictive the Two of Three algorithm classifications were of HIV infection status.

While the prevalence of HIV was lower in Kigali than in Lusaka, we found similar prevalence comparing males and females in each city. This finding disagrees with official data that report two times and four times higher prevalence among young women than in young men in Rwanda and Zambia, respectively [1]. Apart from the fact that these men and women are heterosexual married couples visiting a couple's voluntary counselling and testing site, no other speculations on this difference can be made at this time.

The prevalence of indeterminate/discrepant results among individuals was five-fold higher in Kigali (3.5%) than in Lusaka (0.7%) [6,8,45]. Despite routine trainings and quality assurance programmes, one possible source of the difference is inter-observer variability, particularly in view of the subtlety of faint bands and fine particle agglutination [17,46-48]. SOPs and standard visually based training with photographs of difficult cases is critical to standardize interpretation of rapid tests.

The initial classification was more likely to coincide with the final resolution in "Positive" and "Negative" individuals from Lusaka compared with Kigali, and Lusaka "Other" individuals were more likely to seroconvert. Lusaka individuals were also less likely to have persistent indeterminate/discrepant profiles. This suggests that some causes of false positive rapid test serologies may be more common in Kigali [49-54]. The precise cause is difficult to determine; malaria, syphilis and hepatitis have been associated with false positive HIV serologies [25-29], but all three are less prevalent in Kigali than in Lusaka [55].

The prevalence of pregnancy among women [6,45] was similar in the two samples of couples and has also been proposed as a cause of false positive HIV serologies, but the fact that men were more likely than women to have indeterminate/discrepant results suggests a possible environmental exposure, for example, cattle. Cattle are ubiquitous in Rwanda, where even city dwellers are exposed, while most Lusaka residents are not exposed to cows [49,54] and men are traditionally the cattle herders. Antibodies produced in response to such environmental antigens may interfere with HIV rapid test components based on bovine products.

One final possibility is the nature of the subtypes circulating in these countries and the potential impact on sero-diagnosis. Although the package inserts for all kits used in this study stated that sensitivity and specificity were similar across all African clades, some studies have found that some subtypes may be poorly detected or not identified at all by HIV rapid tests, such as the Determine HIV-1/HIV-2 assay [56].

### "Low HIV-RNA viral load"

The Amplicor HIV-1 Monitor Test was used in cases where follow-up data was not available or did not resolve infection status, and where residual sample was available. Most viral load results were negative, with a small number in low positive range and the rest clearly positive. This test is not licensed for diagnosis and the occasional false positive is not unexpected [57] as samples may not have been handled optimally for molecular testing (e.g., only one tube open at a time, use of screw cap tubes). Potential cross-contamination during sample collection, aliquoting or processing could also contribute to these "low" HIV-RNA viral loads [22,58].

Others have also seen these low values and with subsequent testing have concluded that these individuals were not likely to be infected [58,59]. While all of these issues would still apply, a potential alternative is a PCR-based HIV viral detection test, which is intended for diagnostic use, and is available through perinatal prevention programmes. Where this is available, VCT centres may consider adding collection of cellular material, such as blood spots on filter paper, in addition to serum or plasma.

## Conclusions

Our results support several recommendations for centres using rapid tests for diagnosis of HIV infection:

1. It is important to evaluate algorithms that combine rapid tests.

2. Use of a third rapid test as a tie-breaker does allow point-of-care resolution of most cases of discrepant/indeterminate rapid test results.

a. If two of three tests are negative, the partner is negative, and there are no recent high-risk exposures, routine follow-up testing is not necessary.

b. If two of three tests are positive, follow-up testing is indicated regardless of partner test results or reported exposures.

c. Indeterminate/discrepant profiles that cannot be classified using the Two of Three rule require routine follow-up testing.

3. Routine training of technicians for the visual reading of rapid test results, particularly the more challenging indeterminate results, should take place.

4. Individuals with HIV-infected partners should return in one month for follow-up testing regardless of serologic profile.

5. Most persistent indeterminate/discrepant test profiles do not indicate early HIV infection. They are usually false positive results that may persist for many months, but do not culminate in seroconversion.

6. Complex cases that do not resolve with follow-up testing should be reviewed by a panel of experts and referred as needed for HIV-RNA testing.

7. Strengthen counselling for couples presenting indeterminate or discordant serological results.

8. Counselling messages should be conservative, with a focus on encouraging individuals to return for follow-up testing if indicated. Counsellors should avoid alarming messages, such as, "It is likely that your results indicate early infection with HIV."

9. Improve the standardization of procedures.

## Competing interests

AMC is a member of the Roche Diagnostics: Scientific Advisory Board, Clinical Trial. The other authors have no competing interests to declare.

## Authors' contributions

DIB, SA and EH conceived and designed the experiment. DIB, TS, MK and JB performed the experiments. DIB, NL, SM, AT and SA analyzed the data. AMC contributed reagents. DIB, AMC, SA and EH wrote the paper. EK, EC, CK, KW and WK contributed to participant recruitment and follow-up testing, field site management, protocol development, and manuscript preparation. All authors have read and approved the final manuscript.

## Supplementary Material

Additional file 1 **Table. Frequency distribution of combinations of rapid test results by initial classification**. HIV rapid test results for individuals classified as "Negative", "Other", and "Positive", based on the Two of Three rule. First Response (1^st ^Resp), Determine (Deter), Capillus (Capil), and Unigold (Unig) test results were either clearly negative (-), clearly positive (+), or indeterminate (D). Individuals with final three of three rapid test results negative were resolved as HIV uninfected ("Uninfected"); those with three of three rapid test results positive were resolved HIV infected ("Infected"). Individuals with persistent indeterminate/discrepant rapid test results were finally resolved as "Unresolved". Click here for file
