# Supplementary material for: Indeterminate and discrepant rapid HIV test results in couples' HIV testing and counselling centres in Africa
Source: J Int AIDS Soc. 2011 Apr 8;14:18. doi: 10.1186/1758-2652-14-18 (PMC3086828; doi:10.1186/1758-2652-14-18)
Supplement: Additional file 1 — Table. Frequency distribution of combinations of rapid test results by initial classification. HIV rapid test results for individuals classified as "Negative", "Other", and "Positive", based on the Two of Three rule. First Response (1st Resp), Determine (Deter), Capillus (Capil), and Unigold (Unig) test results were either clearly negative (-), clearly positive (+), or indeterminate (D). Individuals with final three of three rapid test results negative were resolved as HIV uninfected ("Uninfected"); those with three of three rapid test results positive were resolved HIV infected ("Infected"). Individuals with persistent indeterminate/discrepant rapid test results were finally resolved as "Unresolved". [file 1758-2652-14-18-S1.PDF]

					Kigali, Rwanda				Lusaka, Zambia				Total			
	Initial Rapid Test Results				Final Resolution				Final Resolution				Final Resolution			
Two of Three 'Negative'	1st Resp	Deter	Capil	Unig	Total	Neg	Pos	Unres	Total	Neg	Pos	Unres	Total	Neg	Pos	Unres
		D	-	-	120	74	1	45	43	35	0	8	163	109	1	53
	D		-	-	104	73	0	31					104	73	0	31
	+		-	-	55	32	0	23					55	32	0	23
		+	-	-	49	28	1	20	11	9	0	2	60	37	1	22
	-		-	D	11	8	0	3					11	8	0	3
		-	-	D	4	0	1	3					4	0	1	3
	-		D	-	3	1	0	2					3	1	0	2
		-	D	-	4	0	1	3	2	2	0	0	6	2	1	3
	-		+	-	0	0	0	0					0	0	0	0
	-		-	+	2	2	0	0					2	2	0	0
	+	-	-	-	1	1	0	0					1	1	0	0
		-	-	+	1	0	0	1					1	0	0	1
					354	219	4	131	56	46	0	10	410	265	4	141
						62%	1%	37%		82%	0%	18%		65%	1%	34%
	Initial Rapid Test Results				Final Resolution				Final Resolution				Final Resolution			
Two of Three 'Other'	1st Res	Deter	Capil	Unig	Total	Neg	Pos	Unres	Total	Neg	Pos	Unres	Total	Neg	Pos	Unres
		D	-	D	25	9	0	16	4	4	0	0	29	13	0	16
		D	D	-	23	5	0	18	2	2	0	0	25	7	0	18
	D		-	D	28	18	1	9					28	18	1	9
	D		D	-	17	7	1	9					17	7	1	9
		D	D	D	14	8	0	6					14	8	0	6
	+		-	D	8	8	0	0					8	8	0	0
	+		D	-	10	9	0	1					10	9	0	1
		+	D	-	11	9	0	2					11	9	0	2
		D	+	-	7	6	0	1	1	0	1	0	8	6	1	1
	+		D	D	9	7	1	1					9	7	1	1
	D		-	+	10	9	0	1					10	9	0	1
	D		D	D	8	7	0	1					8	7	0	1
	D		D	+	6	6	0	0					6	6	0	0
	D		+	D	7	5	2	0					7	5	2	0
		+	-	D	3	2	0	1	2	1	1	0	5	3	1	1
		+	D	D	5	4	0	1	3	0	3	0	8	4	3	1
	D		+	-	2	2	0	0					2	2	0	0
		-	D	D	1	0	0	1	1	1	0	0	2	1	0	1
	+	-	+	-	1	1	0	0					1	1	0	0
	-		+	D	1	1	0	0					1	1	0	0
	-		D	+	1	1	0	0					1	1	0	0
	-		D	D	0	0	0	0					0	0	0	0
		-	+	D	1	1	0	0					1	1	0	0
		D	+	D	0	0	0	0					0	0	0	0
		D	-	+	1	1	0	0					1	1	0	0
		D	D	+	1	1	0	0					1	1	0	0
	D	-	D	-	1	1	0	0					1	1	0	0
					201	128	5	68	13	8	5	0	214	136	10	68
						64%	2%	34%		62%	38%	0%		64%	5%	32%
	Initial Rapid Test Results				Final Resolution				Final Resolution				Final Resolution			
Two of Three 'Positive'	1st Res	Deter	Capil	Unig	Total	Neg	Pos	Unres	Total	Neg	Pos	Unres	Total	Neg	Pos	Unres
	D		+	+	6	3	3	0					6	3	3	0
		+	-	+					5	2	2	1	5	2	2	1
		+	+	D	4	2	2	0	1	0	1	0	5	2	3	0
	+		+	-	4	4	0	0					4	4	0	0
		+	D	+					2	0	2	0	2	0	2	0
	+		+	D	3	1	2	0					3	1	2	0
		+	+	-	1	1	0	0	3	1	1	1	4	2	1	1
		D	+	+	2	1	0	1	3	0	3	0	5	1	3	1
	+		-	+	1	1	0	0					1	1	0	0
	+		D	+	1	1	0	0					1	1	0	0
	D	+	+	+	1	0	1	0					1	0	1	0
					23	14	8	1	14	3	9	2	37	17	17	3
						61%	35%	4%		21%	64%	14%		46%	46%	8%
